# The horizontal transfer of antibiotic resistance genes is enhanced by ionic liquid with different structure of varying alkyl chain length

**DOI:** 10.3389/fmicb.2015.00864

**Published:** 2015-08-27

**Authors:** Qing Wang, Qian Lu, Daqing Mao, Yuxiao Cui, Yi Luo

**Affiliations:** ^1^School of Environmental Science and Engineering, Tianjin UniversityTianjin, China; ^2^Ministry of Education Key Laboratory of Pollution Processes and Environmental Criteria, College of Environmental Science and Engineering, Nankai UniversityTianjin, China

**Keywords:** antibiotic resistance genes, ionic liquid, horizontal transfer, plasmid, environmental risk

## Abstract

Antibiotic resistance genes (ARGs) have become a global health concern. In our previous study, an ionic liquid (IL) 1-butyl-3-methylimidazolium hexafluorophosphate ([BMIm][PF6]) had been proven to facilitate the dissemination of ARGs in the environment. However, enhanced alkyl group chain length or the substitution of alkyl groups with the cation ring corresponded with increased antimicrobial effects. In this study, we investigated how different structures of ILs with 4, 6, and 8 C atoms in the longer alkyl chain on the imidazolium cations facilitated the dissemination of ARGs. The promotion of plasmid RP4 transfer frequency decreased with [CnMIM][BF4] increasing the alkyl chain length from 4 carbon atoms to 8 carbon atoms on the imidazolium cations, which is observed with [BMIM][BF4] (*n* = 4, 5.9 fold) > HMIM][BF4] (*n* = 6, 2.2 fold) > [OMIM][BF4] (*n* = 8, 1.7 fold). This illustrates that [CnMIM][BF4] with increasing the alkyl chain length exert decreasing ability in facilitating plasmid RP4 horizontal transfer, which is possibly related to IL-structure dependent toxicity. The IL-structure dependent plasmid RP4 transfer frequency was attributable to bacterial cell membrane permeability weaken with increasing alkyl chain length of [CnMIM][PF4], which was evidenced by flow cytometry. In freshwater microcosm, [CnMIm][BF4] promoted the relative abundance of the *sul*I and *int*I genes for 4.6 folds, *aph*A and *tra*F for 5.2 folds higher than the untreated groups, promoting the propagation of ARGs in the aquatic environment. This is the first report that ILs with different structure of varying alkyl chain length facilitate horizontal transfer of plasmid RP4 which is widely distributed in the environment, and thus add the adverse effects of the environmental risk of ILs.

## Introduction

Antibiotic resistance genes (ARGs), as the emerging pollutants, have become a global health concern (Pruden et al., 2006, 2013; Mao et al., 2013; Yang et al., 2013). Horizontal gene transfer rather than the accumulation of point mutations play an important role in the propagation and proliferation of ARGs (Aminov, 2011; Dodd, 2012). The critical role played by plasmids in the horizontal transfer of ARGs has been widely recognized, and plasmid is the main vector for horizontal transfer of ARGs between environmental bacteria (Sørensen et al., 2005; Zhang et al., 2011; Zhu et al., 2013). Plasmid-mediated horizontal transfer of ARGs to indigenous bacteria was previously reported in soil (Henschke and Schmidt, 1990), activated sludge (Merlin et al., 2011), and wastewater oxidation ponds (Mispagel and Gray, 2005). Dissemination and propagation of ARGs via horizontal transfer from antibiotic resistant bacteria to susceptible strains, either between different species or across genera, mainly occur via conjugation of plasmids (DeBruyn et al., 2011; Qiu et al., 2012). Particularly, *in situ* transfer of self-transmissible plasmid RP4 which is a 60-kb broad-host-range conjugative plasmid harbors genes for kanamycin resistance (Km^R^, *aph*A gene), ampicillin resistance (Ap^R^, *tnp*R gene), and tetracycline resistance (Tc^R^, *tet*A gene; Soda et al., 2008).

Ionic liquids (ILs) which are characterized as a big family of compounds with various structures are being considered as “environmental friendly” solvents in modern industry with high polarity and ionic conductivity (Pham et al., 2010), a wide electrochemical window and excellent chemical and thermal stability (Rogers et al., 2002; Zhao et al., 2007). There has not yet been any report of ILs in the environment (Stepnowski, 2005; Markiewicz et al., 2013). However, due to the high water solubility, bioaccumulation, toxicity, and non-biodegradability (Zhu et al., 2009), the ILs have the great potential to increase the risk when they are entering the environment (Zhao et al., 2007; Pham et al., 2010), thus, recent efforts have been toward the potential ecological and environmental risks of ILs (Pham et al., 2010). Adverse effect of some ILs to bacteria are determined by cations (Ganske and Bornscheuer, 2006) and it was found that inhibition effects of *Escherichia coli* cell growth of various ILs change with the number of C atoms in the longer alkyl chain (Lee et al., 2005). Enhanced alkyl group chain length or the substitution of alkyl groups with the cation ring corresponded with increased antimicrobial effects (Docherty and Kulpa, 2005). Recent research also revealed that ILs showed higher antimicrobial activity with increasing alkyl chain length (Pernak et al., 2003; Ranke et al., 2004; Docherty and Kulpa, 2005). In our previous study, we demonstrated that the ILs are capable of facilitating the dissemination of ARGs via horizontal gene transfer (Luo et al., 2014). However, how different structures of ILs with various alkyl chain lengths influence on facilitating the dissemination of ARGs has remained unknown.

In this study, ILs 1-altyl-3-methyl imidazolium tetrafluoroborate ([C_n_MIM][BF_4_], with 4, 6, and 8 C atoms in the longer alkyl chain (as shown in **Table 1**), with different structure of varying alkyl chain length were tested for their potential on horizontal transfer of ARGs mediated by plasmid RP4 from the donors (*E. coli* DH5α) to *Salmonella* (as recipients) in Luria-Bertani (LB) medium and to indigenous bacteria (as recipients) in freshwater microcosms. The effects of bacterial growth and plasmid RP4 horizontal transfer frequency with [CnMIM][BF4] influence of alkyl chain length was determined. Furthermore, alteration of cell membrane permeability with different structure of alkyl chain length by flow cytometry (FCM) was measured to explore a possible mechanism by how the ILs with different structures of alkyl chain length exert different effects on promoting horizontal transfer of plasmid RP4. To our best knowledge, this is the first study of ILs with different structure of varying alkyl chain length facilitating the dissemination of ARGs in both medium of LB and aquatic microcosm.

**Table 1:** Carbon atoms and EC_50_ number of [CnMIM][BF4].

Name	Acronym	Carbon atoms in alkyl chain *(n)*
1-butyl-3-methyl-imidazolium tetrafluoroborate	[BMIM][BF_4_]	4
1-hexyl-3-methyl-imidazolium tetrafluoroborate	[HMIM][BF_4_]	6
1-octyl-3-methyl-imidazolium tetrafluoroborate	[OMIM][BF_4_]	8

## Materials and Methods

### ILs Type

Cationic type of imidazolium (IM) is most widely used in modern industry (Cho et al., 2007; Romero et al., 2008; Zhu et al., 2009) and the 1-alkyl-3-methylimidazolium is one of the most often used IM cations which is attributed to its non-volatility, non-flammability, high thermal stability and an excellent solvent for a wide range of inorganic and organic materials (Gordon, 2001). Anions type of BF4^-^were most widely used in ILs design (Romero et al., 2008).

**FIGURE 1:**
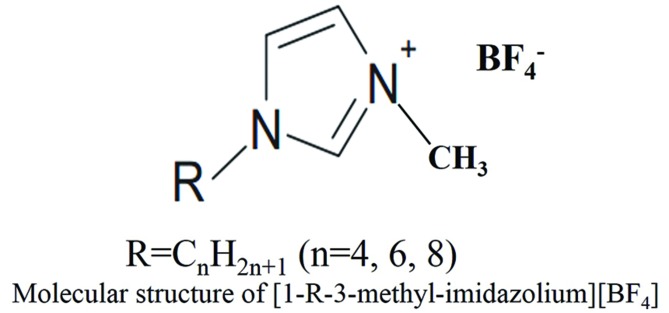
**Molecular structure of 1-R-3-methyl-imidazolium tetrafluoroborate**.

The tested ILs in this study were purchased from Chinese Academy of Sciences (>99% pure). They consist of imidazolium cation and anion of BF_4_^-^, and cation have two alkyl substituents in positions R (**Figure 1**). The ILs 1-alkyl-3-methyl imidazolium tetrafluoroborate used in this study were abbreviated [CnMIM][BF4], where n represent the number of C atoms in the R alkyl chain.

### Setup of Plasmid RP4 Horizontal Transfer in LB System

Plasmid RP4 horizontal transfer in LB system was used to determine the effects of ILs [CnMIM][BF4] on the transfer of plasmid RP4 from *E. coli* DH5α (*E. coli* DH5α, as the donor) to *Salmonella* (Str^R^, as the recipient, across genera). The strain *E. coli* DH5α, harboring the plasmid RP4 carrying ampicillin, kanamycin, and tetracycline resistance genes (Ap^R^, Km^R^, and Tc^R^) but no Str^R^ was used as the donor. The *Salmonella* carrying Str^R^ (in the genome) were used as the recipients and lacked Ap^R^, Km^R^, and Tc^R^. The system was spiked with the ILs [CnMIM][BF4] of initial concentrations of 0, 0.00001, 0.0001, 0.001, 0.01, 0.1, 1.0, 2.5, 5.0, and 10.0 g/L. The IL concentrations in this study were comparable or lower than those in previous toxicity tests (Docherty and Kulpa, 2005; Song et al., 2011; Khudyakov et al., 2012). After mating for 12 h at 30°C, DNA was extracted and then *tra*F gene (indicator for plasmid RP4) and 16S rRNA gene were quantified. Transfer frequency (*f*) was calculated using the formula:

(1) $$f_{1}=t\,r\,a\,F\,g\,e\,n\,e\,\left(c\,o\,p\,i\,e\,s/m\,L\right)/16\,S\,r\,R\,N\,A\,\left(c\,o\,p\,i\,e\,s/m\,L\right)$$

Meanwhile, the number of transconjugants (Ap^R^, Km^R^, Tc^R^, and Str^R^) (N^T^) were counted and the results were presented as colony forming units per milliliter culture (cfu/mL). The number of recipients (Str^R^) (N_S_) was determined by culturing the bacteria on LB agar plates containing 30 mg/L of streptomycin (cfu/mL). Transfer frequency (*f*) was also calculated using the formula:

(2) $$f_{2}=N_{T}\,\left(c\,f\,u/m\,L\right)/N_{s}\,\left(c\,f\,u/m\,L\right)$$

For details on horizontal transfer experiments, please refer to SI-1.

### Setup of Plasmid RP4 Horizontal Transfer in Freshwater Microcosm

Setup of plasmid RP4 horizontal transfer in freshwater microcosms was based on the OECD 308 test (OECD, 2002) to test the effects of ILs [CnMIM][BF_4_] (*n* = 4, 6, 8) on the plasmid RP4 horizontal transfer to indigenous bacteria in the microcosm. The freshwater sample was collected from the Water Parke (39°6′13″N, 117°9′21″E) in Tianjin, China on Sep 2013. Water properties are described in Supplementary Table S1. The strain of *E. coli* DH5α (plasmid RP4) was used as the RP4 donor strain and donor strain *E. coli* DH5α were tracked by specific primer (*E. coli*-FW: GTTCATGTGCCATCTGGTCTT; *E. coli*-RV: AAGTCTTCCTCGGTCGTGAT, Supplementary Table S2). The recipients were indigenous bacteria in freshwater microcosms. Meanwhile, in microcosms, donor isolates were negative in the PCR screening, *tra*F gene (indicator for RP4) and *aph*A gene (resistant gene to kanamycin on RP4) were negative also in the PCR screening. The microcosms were spiked with an IL [CnMIM][BF4] (*n* = 4, 6, 8) of initial concentrations of 0, 0.00001, 0.0001, 0.001, 0.01, 0.1, 1.0, 2.5, 5.0, and 10.0 g/L. Donor-free microcosms also used as controls (no horizontal transfer in donor-free microcosms in pre-experiment). Freshwater microcosms with no added donor and ILs also used as control to exclude mutation. Each concentration was set up in 500 mL of flask and the experiments lasted for 12 h at 30°C, as described in SI-2. Periodic sampling (10 mL) was collected and was used DNA extraction.

### DNA Extraction and Quantitative Polymerase Chain Reaction (qPCR)

Freshwater microcosm samples (5 mL) were centrifuged and the total DNA was extracted using a bacterial DNA Kit according to manufacturer’s instructions (OMEGA, USA). Quantitative polymerase chain reaction (qPCR) assays were used to quantify the 16S rRNA, *sul*1, *int*1, *aph*A, and *tra*F genes. Qualitative PCR assays were conducted in a Biometra T100 gradient thermal cycler (Biometra). The qPCR analyses were performed on a Bio-Rad iQ5 instrument to quantify bacteria 16S rRNA, *sul*1, *int*1, *aph*A, and *tra*F genes. Calibration standard curves for positive controls were generated as previously described. The establishment of negative and positive controls and qPCR reactions were described previously. The primers, amplification details, and standard curves of the 16S rRNA, *sul*1, *int*1, *aph*A, and *tra*F genes are listed in Supplementary Table S2, SI-3 and SI-4.

### Change of Cell Membrane Permeability Measured by Flow Cytometry

To explore the change of cell membrane permeability induced by [CnMIM][BF4] and the relationship between cell membrane permeability and plasmid RP4 transfer frequency in LB system, FCM (BD FACSCalibur) was applied to differentiate and quantify the [CnMIM][BF4]-treated group and control group cells as a function of the percentage of positive cells based on the extent of cell membrane permeability. Propidium iodide (PI; 1 mg/mL; OMEGA, USA) was used to determine increased membrane permeability cells and control cells. Microcosm samples (2 mL) were centrifuged and washed 3x with phosphate buffer solution (PBS). Bacterial cells (the concentration was always less than 10^6^ cells/mL) were then stained at concentrations of 10 μL PI to 1 mL of sample, and incubated in the dark for 8 min before measurement (Xue et al., 2012). The FCM was equipped with an excitation wavelength of 488 nm. All data were processed using CellQuest Pro software (USA).

## Results and Discussion

### Structure Dependent of ILs [CnMIM][BF4] Facilitate Plasmid RP4 Transfer

**FIGURE 2:**
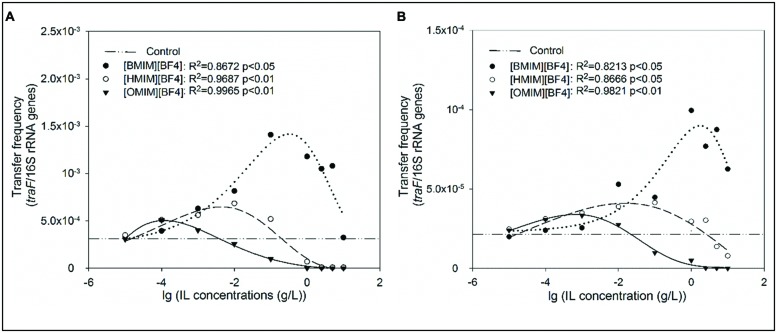
**Influence of alkyl chain length with different concentrations of ILs (0, 0.00001, 0.0001, 0.001, 0.01, 0.1, 1.0, 2.5, 5.0, and 10.0 g/L) on plasmid transfer frequency in freshwater microcosms **(A)** and in LB system **(B)** at mating for 12 h**. The transfer frequency was based on formula (1), *f_1_* = *tra*F gene (copies/mL)/16S rRNA (copies/mL). ([BMIM][BF4], *n* = 4; [HMIM][BF4], *n* = 6; [OMIM][BF4], *n* = 8.)

**FIGURE 3:**
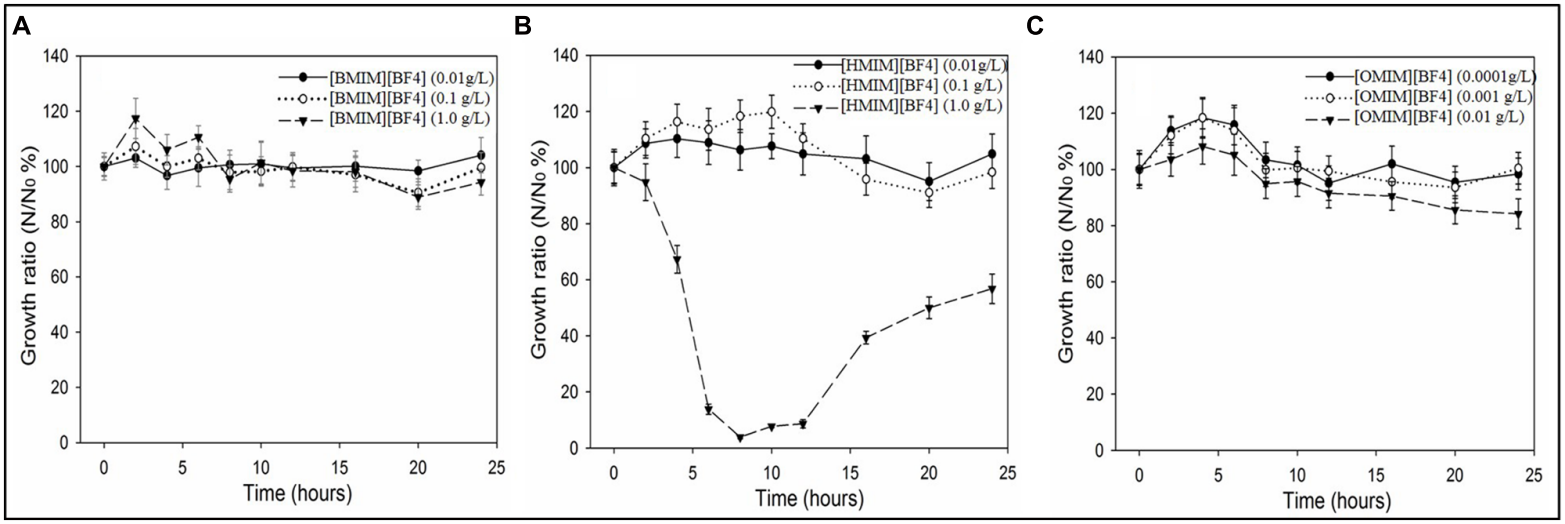
**Growth ratio (N/No, %) of *Escherichia coli* DH5α in LB system treated with [CnMIM][BF4]. (A)** [BMIM][BF4] (concentration of 0.01, 0.1, 1.0 g/L); **(B)** [HMIM][BF4] (concentration of 0.01, 0.1, 1.0 g/L); **(C)** [OMIM][BF4] (concentration of 0.0001, 0.001, 0.01g/L). Control: untreated with [CnMIM][BF4]. ([BMIM][BF4], *n* = 4; [HMIM][BF4], *n* = 6; [OMIM][BF4], *n* = 8).

[CnMIM][BF_4_] (*n* = 4, 6, 8) promoted the plasmid RP4 transfer with dose-effect in freshwater microcosms and LB medium (**Figure 2**). The plasmid RP4 transfer frequency enhanced with increasing [CnMIM][BF4] concentrations, however, decreased when concentration is higher than 1.0 g/L for [BMIM][BF4], 0.01 g/L for [HMIM][BF4], and 0.0001 g/L for [OMIM][BF4]. Meanwhile, the dose-effect dependent of ILs [CnMIM][BF4] facilitating plasmid RP4 transfer associated with ILs’ effects on bacterial growth. In this study, the significant dose-effect inhibition was observed between the bacteria concentrations (the optical densities of OD_600_) and the concentrations of each of the compound ([CnMIM][BF_4_]; **Figure 3** and Supplementary Figure S2B).

Additionally, [CnMIM][BF4] promoted the plasmid RP4 transfer with significant structure-activity both in LB medium and freshwater microcosms (**Figure 2** and Supplementary Figure S1). The promotion of plasmid RP4 transfer frequency decreased with [CnMIM][BF4] increasing the alkyl chain length from 4 carbon atoms to 8 carbon atoms on the imidazolium cations, which is observed with [BMIM][BF4] (*n* = 4, 5.9 fold) > HMIM][BF4] (*n* = 6, 2.2 fold) > [OMIM][BF4] (*n* = 8, 1.7 fold). This illustrates that [CnMIM][BF4] with increasing the alkyl chain length exert decreasing ability in facilitating plasmid RP4 horizontal transfer, which is possibly related to their structure dependent toxicity differences. The size and charge distribution of the individual ions mainly affects the properties of an IL (Wilkes, 2004). Matsumoto et al. (2004) assessed the toxicity of [CnMIM][PF6] (*n* = 4, 6, 8) to the lactic acid-producing of microorganism *Lactobacillus* and found that the production activity of this microorganism decreased with increasing alkyl chain length on the imidazolium cations. Meanwhile, the structure-activity also associated with ILs effects on bacterial growth and enhanced inhibition of bacterial growth with increasing alkyl chain with [CnMIM][BF4] treated in LB system. In this study, the significant structure-activity between the microbial concentrations (the optical densities of OD_600_) and [CnMIM][BF_4_] alkyl chain length in position R were also found (**Figure 3**). The inhibition concentrations of growth of *E. coli*. DH5α for different structures of [CnMIM][BF4] is largely different, it is larger than 2.5 g/L for [BMIM][BF_4_] (*n* = 4), 1.0 g/L for [HMIM][BF4] (*n* = 6), and 0.1 g/L for [OMIM][BF4] (*n* = 8). Additionally, in this study, the ILs inhibition capability for bacterial growth was [BMIM][BF4] (*n* = 4) < [HMIM][BF4] (*n* = 6) < [OMIM][BF4] (*n* = 8) with the same concentration (e.g., 1.0 g/L), which attributed to more toxic of ILs with longer alkyl chains on the cations (Docherty and Kulpa, 2005; Ganske and Bornscheuer, 2006). A relationship between the length of alkyl chain and antimicrobial activities was also found and ILs with short alkyl chain substituent are not active against bacteria and fungi, in contrast ILs containing 10, 11, 12, and 14 carbon atoms in the alkyl chain show very high antimicrobial activities (Pernak et al., 2003; Nancharaiah et al., 2012).

Most interestingly, ILs [CnMIM][BF4] with concentrations of enhancing plasmid RP4 transfer have no influence on bacterial growth. In this study, growth of *E. coli*. DH5α were not influenced when [CnMIM][BF_4_] concentrations are less than 1.0 g/L for [BMIM][BF4], 0.01 g/L for [HMIM][BF4], and 0.0001 g/L for [OMIM][BF4] (**Figure 3**). Consistently, the promotion effect on plasmid RP4 transfer is most obvious when the ILs concentrations are fall in the same range (less than 1.0 g/L for [BMIM][BF4], 0.01 g/L for [HMIM][BF4], and 0.0001 g/L for [OMIM][BF4]). Bacterial concentration plays a crucial role in the plasmid horizontal transfer. In this study, the observed highest values of plasmid transfer frequency were all based on the ILs concentration with no significant inhibition for bacterial growth. Therefore, special attention needs to be paid for ILs [CnMIM][BF4] with concentration especially below the sublethal concentration in the environment, this increase the risk of ILs facilitating the transmission and proliferation of multi-resistance media by plasmid RP4 through horizontal transfer among microorganisms in aquatic environment, and thus posing great risks to public health.

### Structure Dependent of ILs [CnMIM][BF4] Increased Cell Membrane Permeability

**FIGURE 4:**
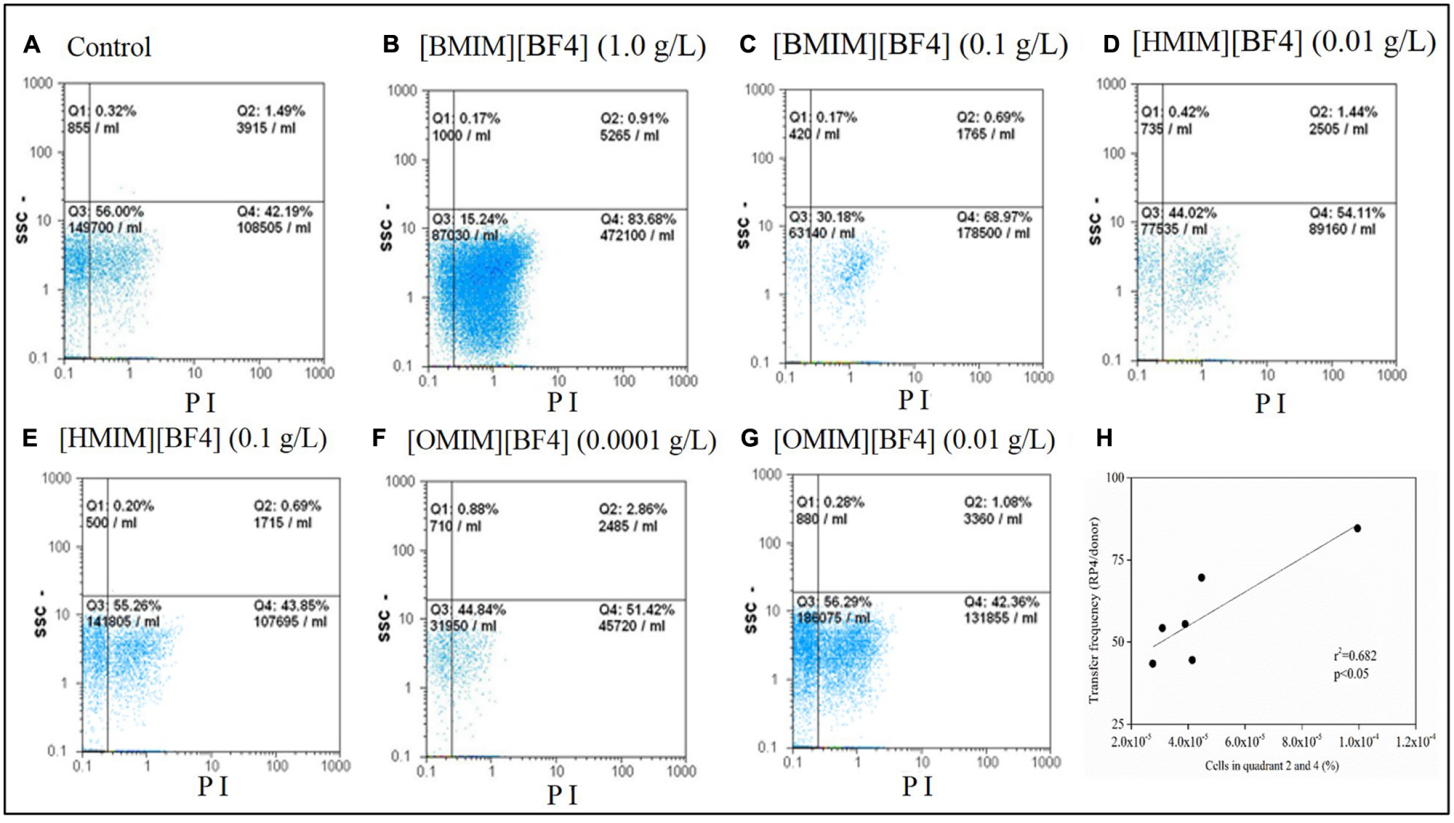
**Cell membrane permeability of microorganisms in LB system treated with [CnMIM][BF4] under the concentration that the highest plasmid RP4 transfer frequency quantified using flow cytometry**. Flow cytometry data in dot plot: Quadrant 1 and 3: negative signal (normal cells); Quadrant 2 and 4: PI positive (cells with increased cell membrane permeability). **(A)** Control: untreated with [CnMIM][BF4]. **(B,C)**: [BMIM][BF4]; **(D,E)**: [HMIM][BF4]; **(F,G)**: [OMIM][BF4]. **(H)** Correlation between transfer frequency in LB system with percentage of cells with increased cell membrane permeability (PI positive) in the total cells treated with [CnMIM][BF4]. ([BMIM][BF4], *n* = 4; [HMIM][BF4], *n* = 6; [OMIM][BF4], *n* = 8.)

A positive correlation (*r*^2^ = 0.682, *p* < 0.05, **Figure 4H**) was found between cell membrane permeability and plasmid RP4 transfer frequency, indicating that enhanced plasmid RP4 transfer could be attributed to increased cell membrane permeability. Meanwhile, FCM assays of microorganisms showed that the percentage of PI-positive cells decreased with increasing alkyl chain length of [CnMIM][PF4], indicating the ability of ILs enhancing bacterial cell membrane permeability weaken with increasing alkyl chain length of [CnMIM][PF4] (**Figure 4**). In the concentration of highest plasmid RP4 transfer frequency, cell membrane permeability increased by 2-fold treated with [BMIM][BF_4_] (*n* = 4; **Figure 4B**, 84.59%) compared with untreated bacteria (**Figure 4A**, 43.58%). The cell membrane permeability increased by 1.27 and 1.24 folds treated with [HMIM][BF_4_] (*n* = 6; **Figure 4D**, 55.55%) and [OMIM][BF_4_] (*n* = 8; **Figure 4F**, 54.28%). The capability of ILs enhancing cell membrane permeability follow the order of [BMIM][BF_4_] (*n* = 4) > [HMIM][BF_4_] (*n* = 6) > [OMIM][BF_4_] (*n* = 8) which is the same as the order of ILs enhancing plasmid RP4 transfer frequency.

Cell membranes are selective semi-permeable membrane and constitute a barrier for horizontal transfer of genetic information among bacteria. Increased cell membrane permeability induced by ILs [CnMIM][PF4] could suppress cell membrane barrier and contribute to genetic information between cells (Thomas and Nielsen, 2005; Alekshun and Levy, 2007). ILs with imidazolium show similar structure to cationic surfactants (Markiewicz et al., 2013), which are known as amphiphilic compounds that contain ionic group and non-polar residues and can increase cell membrane permeability. In this study, the IL [OMIM][BF4] (*n* = 8) contribute to adsorb onto bacterial surface and damage the cell membrane, thereby assisting suppressed cell membrane barrier by increased cell membrane permeability (Thomas and Nielsen, 2005; Alekshun and Levy, 2007). In this study, the maximum horizontal transfer frequency facilitated by 1.0 g/L of [BMIm][BF4] was attributed to the highest cell membrane permeability.

### ILs Facilitated Dissemination of ARGs in Freshwater Microcosms

**FIGURE 5:**
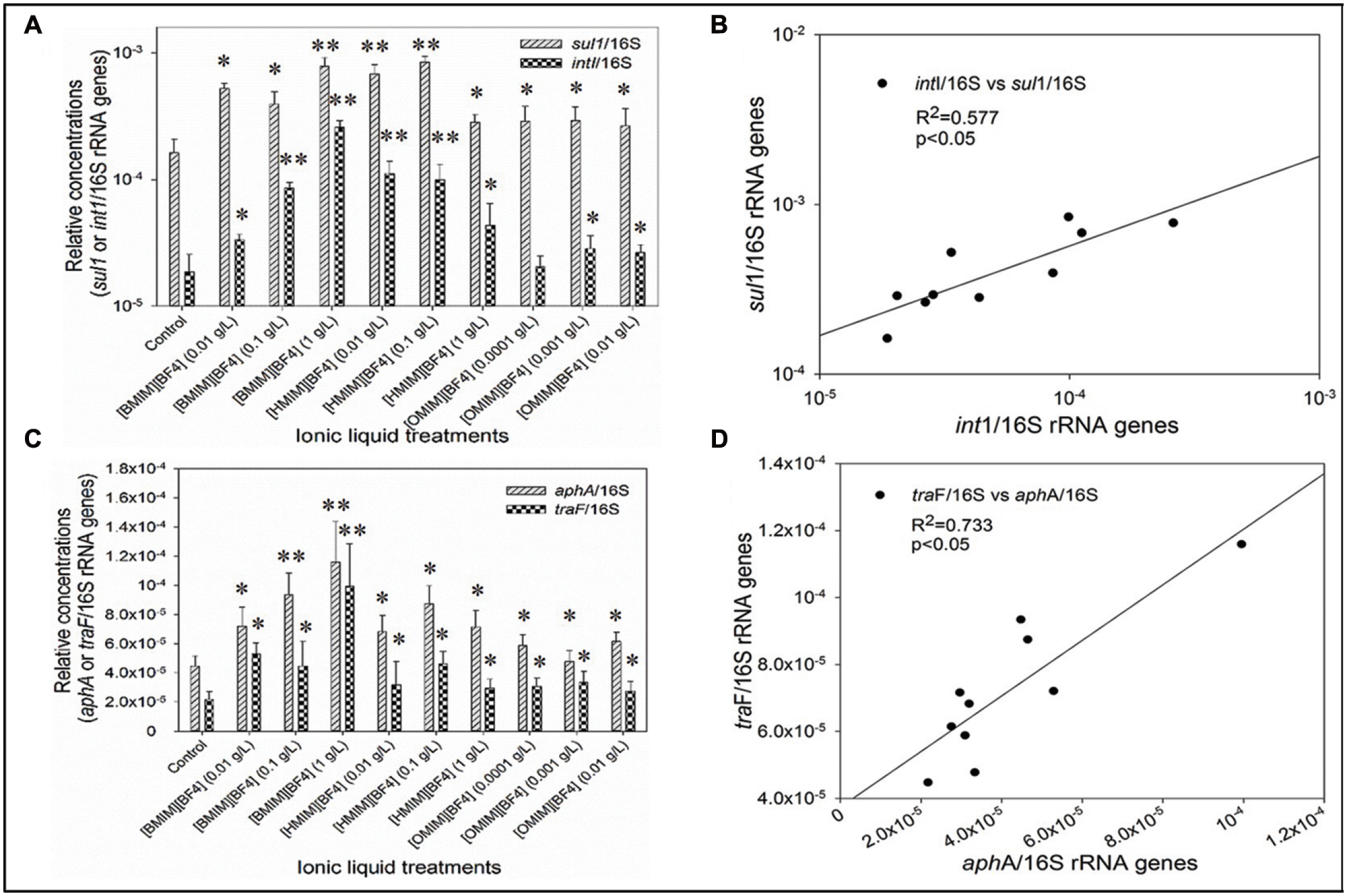
**The relative abundances of *sul*1 gene (*sul*1/16S rRNA) and *int*1 gene (*int*1/16S rRNA; **A**), and correlation between relative abundance of *sul1* gene (*sul*1/16S rRNA) versus relative abundance of *int*1 gene (*int*1/16S rRNA; **B**); the relative abundances of *aph*A gene (*aph*A/16S rRNA) and *tra*F gene (*tra*F/16S rRNA; **C**), and correlation between relative abundance of *aph*A gene (*aph*A/16S rRNA) versus *tra*F gene (*tra*F/16S rRNA; **D**) in the freshwater microcosms treated with [CnMIM][BF4] (0.01, 0.1, 1.0 g/L).** ([BMIM][BF4], *n* = 4; [HMIM][BF4], *n* = 6; [OMIM][BF4], *n* = 8.) The concentration of [CnMIM][BF4] (0.01, 0.1, 1.0 g/L) had a significant effect on the dissemination of ARGs in freshwater microcosms (ANOVA, *p* < 0.05). Significant differences between each of the IL treated groups and the control (0 g/L of IL) were tested with the Student–Newman–Keuls (S–N–K) test, and showed with (^∗^*p* < 0.05) and (^∗∗^*p* < 0.01).

In freshwater microcosms, [CnMIm][BF_4_] (*n* = 4, 6, 8) promoted the relative abundance of the *sul*I (*sul*I gene*/*16S rRNA gene) and *int*I (*int*I gene/16S rRNA gene) genes for 4.6 folds versus that of the untreated controls (**Figure 5A**; *p* < 0.05, S–N–K test). Meanwhile, a positive correlation (*r*^2^ = 0.577, *p* < 0.05) was found between relative abundance of the *sul*I and *int*I genes (**Figure 5B**), implying that the proliferation and propagation of *sul*I gene were primarily attributable to facilitation by class I integrons. The cassette consists of three parts: 5′ conserved segment (5′ CS), 3′ conserved segment (3′ CS) and the variable region between them (Rowe-Magnus et al., 2001). 3′ conserved segment includes two genes that encoding resistance, *sul*I (encoding sulfanilamide resistance) and qacE∆1 (encoding quaternary ammonium salt compounds and ethidium bromine resistance). Variable region harbor different number of gene cassettes, which were used to encode drug-resistance (Sunde, 2005). The propagation of *sul*I, qacE∆1, and ARGs in gene cassette were facilitated by class 1 integrons, which may exhibit enhanced propagation characteristics compared to other mobile genetic elements. Additionally, microbial concentrations (16S-rRNA level) were not significantly change in the microcosm (**Figure 3**). This suggested that rather than bacterial growth, the enhanced the abundance of ARGs was attributable to the dissemination and propagation of ARGs within the cassette of class I integrons.

Meanwhile, [CnMIm][BF_4_] (*n* = 4, 6, 8) facilitated the relative abundance of *aph*A (*aph*A*/*16S rRNA genes) and *tra*F (*tra*F*/*16S rRNA genes), and up to 5.2 folds higher than the untreated controls (**Figure 5C**; *p* < 0.05, S–N–K test). A significant correlation (*r*^2^ = 0.733, *p* < 0.05; **Figure 5D**) was also found between the relative abundance of *aph*A and *tra*F genes, which implies that increased in ARGs were attributable to elevated plasmid RP4 horizontal transfer promoted by ILs [CnMIm][BF_4_]. Meanwhile, [BMIm][PF6] exposure did not influence the donors ratio (Supplementary Figure S2A) and 16S rRNA *ratio* (microbial concentrations, Supplementary Figure S2B), implying that the [BMIm][PF6]-facilitated transfer of class I integrons or plasmid RP4 were not attributed to an increased total bacterial abundance. Plasmids play a critical role in horizontal gene transfer for the spread and dissemination of ARGs in the environment (Zatyka and Thomas, 1998; Sørensen et al., 2005). In this study, plasmid RP4 harbors multi-resistance genes of *aph*A gene (kanamycin resistance), *tnp*R gene (ampicillin resistance), and *tet*A gene (tetracycline resistance; Soda et al., 2008), thus, ILs [CnMIm][BF_4_] exert a different enhanced selective pressure to increase levels of plasmid RP4 through horizontal transfer among microorganism, promoting the propagation of ARGs in the environment. Furthermore, excellent stability makes some ILs conceivable into wastewater treatment plants, and discharged into the aquatic environment (Pham et al., 2010). As consequent, ILs can represent a continuous selective pressure to promote the dissemination and propagation of ARGs.

## Conclusion

In this study, ILs exerts a selective pressure to facilitate the spread of antibiotic resistance through plasmid RP4 horizontal transfer among bacteria, promoting increasing levels of the *sul*I, *int*I, *aph*A, *tra*F genes in aquatic environment. Meanwhile, the peak transfer frequency of promotion of plasmid RP4 transfer were decreased in different degrees with [CnMIM][BF4] (*n* = 4, 6, 8) increasing the alkyl chain length, which attributed to bacterial cell membrane permeability weaken with increasing alkyl chain length of [CnMIM][PF4]. Therefore, in a long period, residual [CnMIM][BF4] in aquatic environment has the great potential to promote the spread of antibiotic resistance. These findings suggest that ILs proposed for use in industrial processes should be carefully evaluated for their ecological and environmental risks before they are discharged into the environment.

## Conflict of Interest Statement

The authors declare that the research was conducted in the absence of any commercial or financial relationships that could be construed as a potential conflict of interest.
