# Supplementary material for: The horizontal transfer of antibiotic resistance genes is enhanced by ionic liquid with different structure of varying alkyl chain length
Source: Front Microbiol. 2015 Aug 27;6:864. doi: 10.3389/fmicb.2015.00864 (PMC4550778; doi:10.3389/fmicb.2015.00864)
Supplement: Supplementary file 1 [file Table_1.DOCX]

**Supplementary materials for**

**The horizontal transfer of resistance genes is enhanced by ionic liquid with different structure of varying alkyl chain length**

*Corresponding author

E-mails: [mao@tju.edu.cn (DQM)](mailto:mao@tju.edu.cn (DQM)), luoy@nankai.edu.cn (YL).

1. horizontal transfer experiment in LB system

Horizontal transfer experiments were used to determine the effects of IL 1-butyl-3-methylimidazolium hexafluorophosphate [BMIm][PF6] on the transfer of plasmid RP4 from *Escherichia coli* DH5α (*E.coli* DH5α, as the donor) to *Salmonella* (Str^R^ as the recipient, across genera). The strain *E.coli* DH5α harboring the plasmid RP4 carrying ampicillin, kanamycin, and tetracycline resistance (Ap^R^, Km^R^, and Tc^R^) and lacking Str^R^ was used as the donor. *Salmonella* carrying Str^R^ (in the genome) were used as recipients, and lacked Ap^R^, Km^R^, and Tc^R^.

The donor and recipient bacteria were grown separately for approximately 14 hours at 37 °C in LB with the appropriate antibiotics. The cultures were centrifuged, followed by three washes with phosphate buffer solution (PBS) (10 g/L NaCl, 0.25 g/L KCl, 1.8 g/L Na_2_HPO4, and 0.3 g/L KH_2_PO_4_), resuspended in LB, and adjusted to specific microbial concentrations (OD_600_=0.400). Then, 2.5 mL of the donor strain and 2.5 mL of the recipient strain were mixed, added to [BMIm][PF6] at concentrations of 0, 0.00001, 0.0001, 0.001, 0.01, 0.1, 1.0, 2.5, 5.0 and 10.0 g/L, and vortexed. These concentrations were established according to exposure concentrations in previous ecotoxicity tests (Docherty and Kulpa, 2005; Stepnowski et al., 2007; Pham et al., 2010; Wells and Coombe, 2006). The cell mixtures were used immediately for plasmid RP4 horizontal transfer experiments.

Transfer experiments were performed in a climate cabinet at 30 °C. After mating for 12 hours, DNA was extracted and then *tra*F gene (indicator for RP4) and 16S rRNA gene were quantified. Meanwhile, the specified cultures were vigorously and 10-fold gradient dilutions were spread on selective plates containing either 100 mg/L of Ap, 50 mg/L of Km, or 10 mg/L of Tc, as well as 30 mg/L of Str. After incubation at 37 °C for 24 hours, the number of transconjugants (Ap^R^, Km^R^, Tc^R^, and Str^R^) (N_T_) were counted and the results were presented as colony forming units per milliliter culture (cfu/mL). The number of recipients (Str^R^) (N_S_) was determined by culturing the bacteria on LB agar plates containing 30 mg/L of streptomycin (cfu/mL). Transfer frequency (*f*) was calculated using the formula (1) and (2) in the manuscript.

In parallel, the donor (plasmid RP4) and recipient bacteria (Str^R^) were also plated onto LB antibiotic plates separately as negative controls to rule out spontaneous mutations of the donor and recipient bacteria.

2. Horizontal transfer experiment in freshwater microcosms

Microcosm horizontal transfer experiments, based on the OECD 308 test (OECD, 2002), were established to determine the effect of Ionic Liquid [BMIm][PF6] on the plasmid RP4 from *E.coli* DH5α on indigenous bacteria in freshwater microcosms. The freshwater sample was collected from the Water Parke in Tianjin, China on Sep 2013. Water properties were described in Table S1. The strain of rifampicin resistance (Rif^R^) *E.coli* DH5α harboring the plasmid RP4, carrying ampicillin, kanamycin and tetracycline resistance (Ap^R^, Km^R^, and Tc^R^), was used as the RP4 donor. The recipients were indigenous bacteria in freshwater microcosms. Meanwhile, in microcosms, donor isolates were negative in the PCR screening, *tra*F gene (indicator for RP4) and *aph*A gene (resistant gene to kanamycin on RP4) were negative also in the PCR screening.

The water samples were stabilized for 3 hours at 4°C in a refrigerator to remove sediment. Supernatant was collected and supplemented with 1% (V:V) Luria-Bertani (LB) media, and incubated overnight on a shaker incubator (160 rpm) at 30°C to reach microbial concentrations (the optical densities) of OD_600_=0.400. Samples were then supplemented with 1% (V:V) *E.coli* DH5α donor strains, making freshwater microcosms.

The microcosm horizontal transfer experiments were set up in a 500 mL flask. Microcosms were spiked with IL [BMIm][PF6] (purity>99%, Chinese Academy of Science, China), resulting in initial freshwater concentrations of 0, 0.00001, 0.0001, 0.001, 0.01, 0.1, 1.0, 2.5, 5.0 and 10.0 g/L, according to previous publications (Docherty and Kulpa, 2005; Stepnowski et al., 2007; Pham et al., 2010; Wells and Coombe, 2006), and mixed by vortexing.

The microcosm horizontal transfer experiments were stabilized in a climate cabinet at 30°C for 24 h. 10 mL of microcosm samples were sampled for DNA extraction.

3. PCR and qPCR conditions of target genes

Qualitative PCR assays were conducted in a Biometra T100 gradient (Biometra, Germany) as follows (25 μL): The PCR procedure for an initial DNA denaturation (95°C for 5 min), followed by 30 cycles of 30 sec at 95°C (denaturing), 30 sec of annealing at the temperatures specified in Table S2, and 1 min at 72°C (extension), followed by a final extension of 7 min at 72°C.

qPCR analyses were performed on a Bio-Rad iQ5 instrument (Bio-Rad Company, CA, USA). Amplification (25 μL) consisted of an initial cycle of initial denaturing 5 min at 95℃, followed by 40 cycles of 15 s at 95℃, 1 min at annealing temperatures (Table S2), 30 s at 72℃ and a final melt curve stage with temperature ramping from 55℃ to 95℃(0.5℃ per read, 30 s hold) (Luo et al., 2010).

4. Standard curves

Standard curves were established before qPCR analyses on a Bio-Rad iQ5 instrument (Bio-Rad Company, CA, USA) to quantify 16S rRNA, *sul*1, *int*1, *aph*A, and *tra*F genes. After normal PCR amplification, fresh PCR products of 16S rRNA, *aph*A, and *Tra*F genes were recovered, purified and ligated onto a pEASY-T3 vector (TransGen, China) and transformed to *E.coli* DH5α according to manufacturer’s instructions (TransGen, China). Positive clones were screened by blue-white selection, and PCR and sequencing were used to verify cloning of the target genes. Plasmids carrying target genes were chosen as the standards for qPCR. Plasmids carrying target genes were extracted according to the plasmid extraction kit (OMEGA, USA). The concentration and quality of the plasmid were determined by spectrophotometric analysis and agarose gel electrophoresis. The copy number of target genes per microliter of plasmid solution was calculated as previously described (Zhang et al., 2009). Eight-point standard curves for qPCR were generated using 10-fold serial dilutions of the plasmid carrying target genes. R^2^ values were higher than 0.990 for all standard curves. According to the standard curves, the Ct value of samples was used to calculate the gene copies of 16S rRNA, *sul*1, *int*1, *aph*A, and *tra*F genes.

5. Statistical analysis

Data analysis was conducted using SPSS Statistics Version 20.0 (IBM, NY, USA). The correlation between transfer frequency in LB system with percentage of cells with increased cell membrane permeability (PI positive) in the total cells treated with [CnMIM][BF4], correlation between relative abundance of the *sul*1 (*sul*1 gene /16SrRNA gene) and *int*1 (*int*1 gene /16SrRNA gene) genes, and correlation between relative abundance of the *aph*A (*aph*A gene /16S rRNA gene) and *tra*F (*tra*F gene /16S rRNA gene) genes were analyzed using a Pearson correlation analysis. The averages and standard deviations of the data were calculated using Excel 2007 (Microsoft Inc., USA). OriginPro 9.0 (Origin Lab Corporation, USA) was used for plotting.

5. The properties of water quality in samples

Analysis of antibiotics was conducted using HPLC-MS/MS, and details including Sample pretreatment, solid phase extraction and HPLC MS/MS Analysis are described in our previous study (Luo et al., 2010).

Heavy metals were quantified using inductively coupled plasma mass spectrometry (ICP-MS) (Sciex Elan DRC-e, USA).

TOC analysis used a TOC analyzer (analytikjena multi N/C 3100, Germany), NH3-N analysis used ammonium-Nessler’s reagent spectrophotometry. Dissolved phosphorus (dP) was analyzed by Mo-Sb antispectrophotometry method.





**Figure S1. Influence of alkyl chain length with different concentrations of ILs (0, 0.00001, 0.0001, 0.001, 0.01, 0.1, 1.0, 2.5, 5.0 and 10.0 g/L) on plasmid transfer frequency in LB system at mating for 12 hours.** **The** **transfer frequency was based on formula (2), *f* = N_T_ (cfu/mL)/N_S_ (cfu/mL).** ([BMIM][BF4], n= 4；[HMIM][BF4], n=6; [OMIM][BF4], n=8).


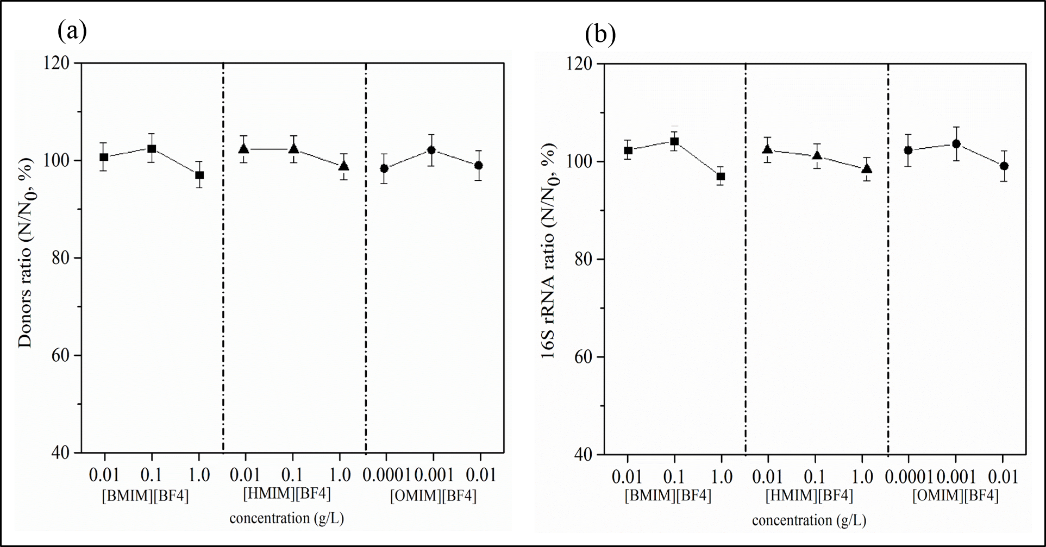


**Figure S2. Donors ratio (N/No, %) of *E. coli* DH5α (a) and 16S rRNA ratio (N/No, %) (b) in freshwater microcosms system treated with [CnMIM][BF4] at mating for 12 hours. [BMIM][BF4] (Concentration of 0.01, 0.1, 1.0 g/L); [HMIM][BF4] (Concentration of 0.01, 0.1, 1.0 g/L); [OMIM][BF4] (Concentration of 0.0001, 0.001, 0.01g/L). Control: untreated with [CnMIM][BF4]**. ([BMIM][BF4], n= 4；[HMIM][BF4], n=6; [OMIM][BF4], n=8).

**Table S1.** Freshwater properties of samples collected from the Water Parke in Tianjin, China

| Test items | Measurements |
| --- | --- |
| Enrofloxacin (ng/L) | 36.0 |
| Tetracycline (ng/L) | 300.4 |
| Sulfamethazine (ng/L) | 0.4 |
| Ciprofloxacin (ng/L) | 26.8 |
| Ofloxacin (ng/L)  kanamycin | 10.0  N.D* |
| Oxytetracycline (ng/L) | 11.6 |
| Sulfamethoxazole (ng/L) | 82.4 |
| Doxycycline (ng/L)  Streptomycin (ng/L) | 5.2  50.5 |
| Chlortetracycline (ng/L) | 16.8 |
| Roxithromycin (ng/L)  Ampicillin  Rifampicin | 7.2  N D  N D |
| Zinc (ug/L) | 50.0 |
| Copper (ug/L)  TOC (mg/L)  NH3-N (mg/L)  Dissolved P (mg/L) | 10.0  16.8  3.2  0.2 |
| pH | 7.2 |
| Water temperature (°C) | 30 |

* ND, not detected.

**Table S2.** PCR primers and PCR conditions

| Primer | Target | Sequence (5’-3’) | PCR annealing temp (ºC) | qPCR annealing temp (ºC) | Amplicon size (bp) | Source |
| --- | --- | --- | --- | --- | --- | --- |
| 16s-FW | 16S  rRNA | CGGTGAATACGTTCYCGG | 58 | 57.5 | 126 | (Suzuki et al., 2000) |
| 16s-RV |  | GGWTACCTTGTTACGACTT |  |  |  |  |
| 27F | 16S  rRNA | AGAGTTTGATCCTGGCTCAG | 56 | - | 1466 | (Lane, 1991) |
| 1492R |  | GGTTACCTTGTTACGACTT |  |  |  |  |
| *aph*A-FW | *aph*A | GGCTTCGTGATGCCTGCTT | 62 | 62 | 198 | This study |
| *aph*A-RV |  | CATTCCTGGCCGTGGTTCT |  |  |  |  |
| *tra*F-FW | *traF* | CTCCGATGGAGGCCGGTAT | 54.1 | 54.1 | 196 | This study |
| *tra*F-RV |  | GGGAATGCCATCTGCCTTGA |  |  |  |  |
| *Int*1-FW | *class I*  *integrons* | GGCTTCGTGATGCCTGCTT | 57 | 55 | 145 | This study |
| *Int*1-RV |  | CATTCCTGGCCGTGGTTCT |  |  |  |  |
| *sul*1-FW | *sul*1 | CACCGGAAACATCGCTGCA | 57.5 | 60 | 157 | This study |
| *sul*1-RV |  | AAGTTCCGCCGCAAGGCT |  |  |  |  |
| *E.coli*-FW | *E.coli* | GTTCATGTGCCATCTGGTCTT | 55.5 | 55.5 | 214 | This  study |
| *E.coli*-RV |  | AAGTCTTCCTCGGTCGTGAT |  |  |  |  |

*FW, forward; RV, reverse.

**REFERENCES**

Docherty, K. M. and C. F. Kulpa (2005). "Toxicity and antimicrobial activity of imidazolium and pyridinium ionic liquids." Green Chem **7**(4): 185-189. DOI: 10.1039/b419172b.

Lane, D. (1991). "16S/23S rRNA sequencing." Nucleic acid techniques in bacterial systematics.

Luo, Y., D. Q. Mao, M. Rysz, Q. X. Zhou, H. J. Zhang, L. Xu and P. J. J. Alvarez (2010). "Trends in antibiotic resistance genes occurrence in the Haihe River, China." Environ Sci Technol **44**(19): 7220-7225. DOI: 10.1021/es100233w.

OECD (2002). "Aerobic and Anaerobic Transformation in Aquatic Sediment Systems. In Guidelines for Testing of Chemicals No 308." Organisation for Economic Cooperation and Development.

Suzuki, M. T., L. T. Taylor and E. F. DeLong (2000). "Quantitative analysis of small-subunit rRNA genes in mixed microbial populations via 5′-nuclease assays." Appl Environ Microb **66**(11): 4605-4614. DOI: 10.1128/AEM.66.11.4605-4614.2000.

Stepnowski, P., W. Mrozik and J. Nichthauser (2007). "Adsorption of alkylimidazolium and alkylpyridinium ionic liquids onto natural soils. " Environ Sci Technol **41**(2): 511-516. DOI: 10.1021/es062014w.

Pham,T.P., C. W. Cho and Y. S. Yun (2010). "Environmental fate and toxicity of ionic liquids: A review." Water Res **44**(2): 352-372. DOI:10.1016/j.watres.2009.09.030.

Wells, A. S. and V. T. Coombe (2006). "On the freshwater ecotoxicity and biodegradation properties of some common ionic liquids." Org Process Res Dev **10**(4): 794-798. DOI: 10.1021/op060048i.

Zhang, T., M. Zhang, X. Zhang and H. H. Fang (2009). "Tetracycline resistance genes and tetracycline resistant lactose-fermenting Enterobacteriaceae in activated sludge of sewage treatment plants." Environ Sci Technol **43**(10): 3455-3460. DOI: 10.1021/es803309m.
